# Do People Eat the Pain Away? The Effects of Acute Physical Pain on Subsequent Consumption of Sweet-Tasting Food

**DOI:** 10.1371/journal.pone.0166931

**Published:** 2016-11-18

**Authors:** Kathleen E. Darbor, Heather C. Lench, Adrienne R. Carter-Sowell

**Affiliations:** Department of Psychology, Texas A&M University, College Station, Texas, United States of America; Université catholique de Louvain, BELGIUM

## Abstract

Sweet tasting foods have been found to have an analgesic effect. Therefore people might consume more sweet-tasting food when they feel pain. In Study 1, participants were randomly assigned to a pain or non-pain condition and their consumption of cheesecake was measured. Participants ate more cheesecake (a sweet-tasting food) following a painful experience than a non-painful one. In Study 2, participants were randomly assigned to a painful experience or a resource depleting experience (i.e., squeezing a handgrip) and then were asked to taste test two foods, one sweet and one not sweet. Participants ate more sweet-tasting food following a painful experience than a non-painful or a resource-depleting experience. These differences were not present for consumption of non-sweet food. Further, habitual self-control predicted consumption of sweet-tasting food when in pain, with those lower in self-control particularly likely to eat more. Results suggest that people do eat more sweet-tasting food when they feel pain, particularly if they are not in the habit of controlling their impulses. These findings have implications for health given rising rates of obesity and pain-related diagnoses.

## Introduction

Excessive consumption of sweet-tasting foods is arguably an impulsive behavior with large implications for human health. There is growing worldwide concern about obesity and related health problems, including diabetes, heart disease, and certain types of cancer [1]. In the last two decades, obesity has become one of the leading contributors to mortality in developed nations. It is estimated that obesity costs the United States alone nearly $73.1 million per year in productivity and $209.7 billion per year in health care [2, 3]. If physical pain, an almost daily experience, increases consumption of sweet-tasting foods (which often contain high amounts of added sugar), there could be serious health and economic consequences.

Physical pain has typically been approached from a biological perspective, with relatively little experimental work available on the behavioral and cognitive consequences of physical pain. In contrast, the consequences of social pain have been extensively explored in the last several decades. Social pain represents an unpleasant emotional response that occurs in reaction to separation from people or groups, and has been associated with multiple changes in behavioral and cognitive outcomes [4, 5]. Although physical and social pain are not synonymous, they both engage similar neural circuitry [5, 6, 7] and both types of pain can result in aggression and temporary numbness to subsequent physical pain [8, 9]. Such findings have resulted in theoretical conceptualizations that social pain and physical pain overlap in the sense that they share similar antecedents and consequences [4, 9–11].

One of the more serious consequences of pain is its potential to impair choice. The tendency to rely on impulse to make choices, rather than systematic analysis, underlies numerous behaviors with individual and societal impacts, including addiction, gambling, overeating, and poor health behavior [12–14]. Social pain has been demonstrated to increase a variety of poor choices related to impulsive processing, including consumption of unhealthy foods, preferences for immediate gratification, and procrastination [8, 15, 16]. One explanation for the impact of social pain on choice is that the experience of pain depletes resources that are required for self-control, resulting in people giving in to their impulses [17]. According to the resource model of self-control, the resources required to regulate behavior are limited and consequently people will make poor choices when their regulatory resources have been depleted (e.g., [18]). Consistent with the proposition that social pain impairs behavior because of reduced regulatory resources, people high on social anxiety, who are particularly likely to devote limited resources to rumination about social situations, consumed more of an unhealthy food than others after being socially rejected [19].

Because of the overlap between social and physical pain, there is reason to expect that physical pain will similarly impair choices related to impulsive processing. As with social pain, there is some evidence that chronic and acute physical pain can increase preferences for immediate gratification [20–22]. Impaired choice on tasks related to impulsive processing could result from physical pain as a result of overburdened regulatory resources, particularly for chronic pain [4], although this possibility has not been directly tested. However, there are reasons to expect that the effects of *acute* physical pain on choice could be driven by a process other than reduced regulatory resources.

Although social and physical pains have many similar antecedents and consequences, there is an important distinction between them that could result in different processes driving choice following the experience of acute pain. People devote cognitive resources to ruminating about social pain in a manner that they do not ruminate about physical pain [19], and thinking about past social pain reinstitutes the experience of pain whereas thinking about past physical pain does not [23, 24]. Thus, while acute physical pain might commandeer attention as it is experienced, these demands would typically reduce immediately after the end of the painful experience [25]. Because of this reduction in regulatory demand after the end of a painful experience, acute physical pain might not impair choice because of depleted regulatory resources.

Indeed, there is an alternative process that is likely to impair choices related to food consumption following acute physical pain. Pain could impair choice because it alters the value assigned to sweet-tasting food. There is evidence that consumption of sweet-tasting food (i.e., high in sucrose), actually does impact the experience of pain. For example, consumption of sweet-tasting foods increases pain tolerance through endogenous opioid activity in the brain [26–28], and opiate receptor antagonists (e.g., naloxone) can reduce the hedonic value of sweet-tasting foods [26]. As a result of this connection between consumption of sweet-tasting foods and pain reduction [29], the value assigned to foods high in sucrose might increase in conjunction with the experience of acute physical pain, with people enacting this heightened value by consuming more sweet-tasting food after pain experiences. Interestingly, evidence suggests that pain could increase consumption without altering self-reported liking of the stimuli. People demonstrate increased motivation to attain rewards during pain, but self-reports of liking are not changed [30], and after the experience of pain the typical relationship between self-reported liking of a food and consumption is disrupted [31]. The possibility that recent pain experience could change the hedonic value of unhealthy foods is also consistent with predictions derived from prospect theory, in that the value placed on outcomes is relative to one’s current standing [32]. Accordingly, it might be that acute physical pain results in greater consumption because people place greater value on the sweet-tasting food.

The present investigation is the first to examine the effects of acute physical pain on consumption. Based on findings that pain can increase impulsive decisions and that sweet-tasting food has analgesic properties, we predicted that acute physical pain would increase consumption of sweet-tasting food.

## Study 1 Materials and Methods

Undergraduates in introductory psychology courses were prescreened at the beginning of the semester for circulatory disorders and liking of cheesecake, rated on a scale from 1 (*hate*) to 5 (*love*). Those who reported no history of circulatory disorders (who could be injured by the pain task), and who did not dislike or hate cheesecake (which would influence the outcome measure), were invited to participate (*n* = 70; 18.61 years old, 65% women). A power analysis revealed that this sample size had sufficient power (1-β = .95) to detect medium to large effects (.04). The anticipated effect size was consistent with prior investigations on the effects of negative emotion on consumption [19]. Study and consent procedures were approved by the Texas A&M University IRB. No minors were included in any study. Participants provided written consent to participate in all studies. Data for studies is available at https://osf.io/vz7y4/. Videos are not publicly accessible because participants are identifiable.

Pain was induced through a cold pressor task, a common method of inducing pain that involves immersion of the hand in cold water, creating an aching or crushing pain that rapidly increases in intensity [33]. Participants were randomly assigned to place their non-dominant hand in a bucket of either room temperature or freezing water (held at 0 degrees Celsius; conditions ran so that the same water temp could be used in session blocks). They were asked to leave their hand in the water for two minutes (five participants withdrew their hands early but were included in analyses because the manipulation of pain, although shorter, was effective). At the end of the two minutes they rated their current level of pain on the Numeric Pain Intensity Scale (NPIS) from 0 (*no pain*) to 10 (*worst possible*), and on the Wong-Baker FACES scale from 0 (*no hurt*) to 5 (*hurts worst*) [34]. The pain manipulation was effective, as participants felt more pain on the NPIS in the pain condition (*M* = 5.09, *SD* = 2.49) than in the control condition (*M* = 0.50, *SD* = 0.92), *Levene's F* = 18.51, *p* < .001, *t*(42.21) = 10.03, *p* < .001, *d* = 2.47, 95% CI [1.75, 3.18] and on the FACES scale (*M*_*pain*_ = 2.58, *SD* = 1.18, *M*_*control*_ = 0.16, *SD* = 0.37), *Levene's F* = 33.21, *p* < .001, *t*(36) = 10.91, *p* < .001, *d* = 2.69, 95% CI [1.90, 3.46].

Immediately after rating their pain, participants were given an opportunity to consume a sweet-tasting food–cheesecake—and they were led to believe this was not part of the experiment. They were told there was a delay in setting up the next task and they could have a slice of cheesecake while they waited. If they refused the cheesecake, the experimenter told them they would leave it on the table anyway, in case the participant changed his/her mind. The participant was then left alone with the cheesecake and a fork for 6 minutes (pilot testing indicated that if participants did not consume the cake within this time frame, they were unlikely to do so later). The weight of the cheesecake was measured on a digital scale before and after the study. Following the standard used in previous studies that measured consumption by weight using similar methodology [19], weight difference scores were transformed by rank using Blom’s formula to correct for skew (the inferences that can be made from analyses remain identical without this correction). While alone in the room, participants were surreptitiously video recorded for later coding of number of bites taken by two independent coders blind to pain condition (*r* = .96). At the conclusion of the study, participants rated how motivated they were to resist eating the cheesecake on a scale ranging from 1 (*extremely unmotivated*) to 7 (*extremely motivated*), and completed a suspicion check and process debriefing. In general, participants reported that they were motivated to resist eating the cheesecake (*M* = 3.50, *SD* = 2.07), compared to a rating of four indicating no motivation, *t*(61) = 1.90, *p* = .062, *d* = .24, 95% CI [-.01, .49], suggesting they were trying not to consume this sweet-tasting food. Five participants reported suspicion that the cheesecake was part of a study on pain, and they were retained in the interest of being inclusive (results were similar without them).

## Study 1 Results and Discussion

Participants in the pain condition ate significantly more cheesecake based on the weight difference before and after (*M* = 0.30, *SD* = 0.16) than participants in the control condition (*M* = 0.20, *SD* = 0.17), *t*(68) = 2.38, *p* = .020, *d* = .57, 95% CI [.09, 1.05]. Participants in the pain condition also took significantly more bites of cheesecake (*M* = 0.20, *SD* = 0.12) than participants in the control condition (*M* = 0.13, *SD* = 0.13), *t*(56) = 2.01, *p* = .049, *d* = .53, 95% CI [.002, 1.05]. These results demonstrate that the experience of acute physical pain can increase consumption of sweet-tasting food.

## Study 2

The obvious question that arises from the findings of Study 1 is whether the effects of pain are unique to the consumption of sweet-tasting food. To find out, consumption of both sweet and non-sweet food was examined in Study 2. A secondary question that arises from the findings is: why are people eating more sweet-tasting food when in pain? There are two alternative explanations for increased consumption following acute physical pain: 1) mood improvement, or 2) impaired regulation. From a mood improvement perspective, the experience of pain would increase consumption of sweet-tasting foods because people believe eating such foods will make them feel better [16]. This expectation is particularly likely for consumption of sweet-tasting foods, given that they actually have analgesic properties. In contrast, from an impaired regulation perspective, holding one’s hand in painfully cold water and curtailing the consumption of sweet-tasting foods both require limited regulatory resources [8, 35]. If the pain manipulation depleted these resources, it would leave people unable to resist the temptation of sweet-tasting foods. Study 2 included a self-report scale of emotional eating and a depletion condition to tease apart the importance of expected mood improvement or resource depletion in consumption of sweet-tasting food. We also included a measure of trait self-control to capture differences in the tendency to exert control over impulses. Hypotheses for this study were preregistered at osf.com. Study and consent procedures were approved by the Texas A&M University IRB. No minors were included in any study. Participants provided written consent to participate in all studies. Data for studies is available at https://osf.io/vz7y4/. Videos are not publicly accessible because participants are identifiable.

## Study 2 Materials and Methods

Undergraduates participated in the study (*n* = 159; 18.42 years old, 74% female). Rather than prescreen for circulatory disorders, participants who indicated such an issue were reassigned to a non-pain condition if necessary. The sample size was set in advance at 150 participants (50 per condition), and data collection was terminated as soon as possible after this number was reached.

Participants were randomly assigned to one of three conditions: the pain condition involving the cold pressor task described in Study 1, the no pain condition involving the warm water task in Study 1, or the depletion condition. In the depletion condition, participants held a hand-squeeze device for two minutes, with a foam ball held in the handle so it would be evident if they released their grip [36]. This manipulation has been used previously to induce depletion of regulatory resources [18, 36]. Participants rated their pain or strain (in the depletion condition; scale points were changed to reflect “strain” instead of “pain”) afterward. Preliminary analyses revealed a main effect of condition on the NPIS ratings and the FACES scale, consistent with an effective manipulation, *F*(1, 155) = 107.19, *p* < .001, η^2^ = .58, and *F*(1, 155) = 138.78 *p* < .001, η^2^ = .64, respectively. Participants reported more pain on the NPIS and the FACES in the pain condition (*M*_*NPIS*_ = 4.93, *SD* = 2.30; *M*_*FACES*_ = 2.84, *SD* = 1.14) than in the no pain condition (*M*_*NPIS*_ = 0.15, *SD* = .45; *M*_*FACES*_ = 0.07, *SD* = 0.26), *Levene's F* = 86.40, *p* < .001, *t*_*NPIS*_(54.80) = 14.75, *p* < .001, *d* = 2.85, 95% CI [2.19, 3.50], *Levene's F* = 57.11, *p* < .001, *t*_*FACES*_(56.27) = 17.03, *p* < .001, *d* = 3.29, 95% CI [2.57, 4.00]. Participants also reported more strain on the NPIS and FACES in the depletion condition (*M*_NPIS_ = 2.64, *SD* = 1.77; *M*_*FACES*_ = 1.44, *SD* = 0.92) than pain in the no pain condition, *Levene's F* = 59.80, *p* < .001, *t*_*NPIS*_(57.41) = 9.88, *p* < .001, *d* = 1.93, 95% CI [1.04, 2.44], and *Levene's F* = 81.73, *p* < .001, *t*_*FACES*_(59.14) = 10.36, *p* < .001, *d* = 2.02, 95% CI [1.49, 2.55].

Consumption was assessed through a "taste test" paradigm, in which participants were asked to provide ratings of the qualities of two foods. The foods were skittles (sweet-tasting option) and raisins (less sweet option), and participants were encouraged to eat as much as needed to make their judgments. The bowls containing the food items were weighed before and after the taste test task, and participants were instructed that they would have 6 minutes to make their judgments. After the taste test, participants completed questionnaires on a computer, including demographics questions (gender, age), the Emotional Eating Scale [37], the Brief Self-Control Scale [38], and the Ten Item Personality Inventory (TIPI) [39].

## Study 2 Results

**Fig 1:**
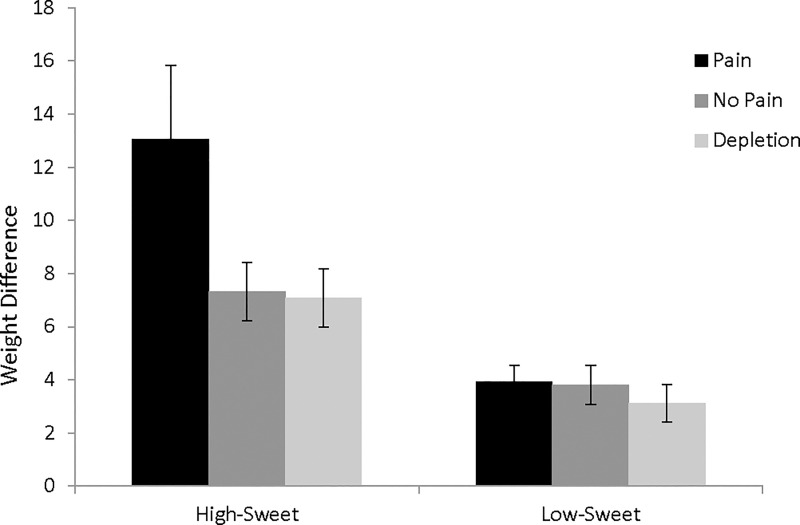
Participants in pain consumed more sweet-tasting food than participants in the no pain or depletion conditions.

A mixed ANOVA was conducted with condition (pain, no pain, depletion) as the between-subject factor and differences in grams of each food type consumed (high-sweet, low-sweet) as the repeated measure. Results revealed a marginal main effect of condition, *F*(1, 155) = 2.94, *p* = .056, *η*^2^ = .036, and a main effect of food type, *F*(1, 155) = 29.67, *p* < .001, *η*^2^ = .16. These main effects were qualified by a significant interaction, *F*(1, 155) = 3.15, *p* = .046, *η*^2^ = .039. As shown in Fig 1, participants who were in pain consumed more high-sweet food than participants in the no pain condition, *Levene's F* = 7.29, *p* = .008, *t*(68.16) = 1.92, *p* = .059, *d* = 0.37, 95% CI [-.01, .75], or the depletion condition, *Levene's F* = 7.99, *p* = .006, *t*(67.94) = 1.95, *p* = .056, *d* = .38, 95% CI [-.01, .76]. The pain condition did not differ from the other conditions in consumption of low-sweet food, *t*(105) = 0.13, *p* = .893, *d* = .03, 95% CI [-.35, .40], and *t*(102) = 0.89, *p* = .374, *d* = .17, 95% CI [-.21, .55]. Overall, the results support an account that participants in pain consume more sweet-tasting food in particular.

As can be seen in Fig 1, there was also greater variability in participants' consumption of sweet-tasting food in the pain conditions than in the other conditions or for the non-sweet food option. We therefore also investigated two potential moderators of consumption of sweet-tasting foods when in pain—the tendency to engage in emotional eating and trait differences in self-control. Because we were only interested in explaining variation in consumption in the pain condition, we limited analyses to this condition, and the moderator variables were entered into separate linear regressions as predictors of the amount of sweet-tasting food consumed. Self-reported tendency to engage in emotional eating did not predict consumption in the pain condition, β = .15, *t* = 1.08, *p* = .285. Trait self-control marginally predicted consumption in the pain condition, β = -.27, *t* = -1.99, *p* = .052, with lower trait self-control predicting greater consumption of sweet-tasting food.

## Conclusions

Like social pain, acute physical pain impaired choice in the present investigation in the sense that it resulted in increased consumption of sweet-tasting food that participants reported they desired to avoid eating (e.g., [19]). This finding may have important implications for public health, given that physical pain is a common daily occurrence and that there are many people living with chronic pain which is accompanied by periods of acute pain [40]. If physical pain, an almost daily experience, increases consumption of sweet-tasting, frequently unhealthy, foods, there could be serious health and economic consequences. There is growing worldwide concern about obesity and related health problems, including diabetes, heart disease, and certain types of cancer [1], costing billions per year in lost productivity and health care [2, 3].

The results were not consistent with accounts that people consume more following pain because they have depleted their regulatory resources, as consumption was not increased in the depletion condition and was specific to sweet-tasting food. Instead, a self-report of the degree to which participants habitually controlled impulses predicted consumption of sweet-tasting food when in pain. Thus, people who have lower habitual self-control consumed more of a sweet-tasting food, suggesting that those high in self-control can resist the temptation to eat sweet-tasting food even when experiencing acute physical pain. Increased consumption of sweet-tasting food when in pain could be driven solely by changes in psychological motivation, but it is possible that changes in the value placed on sweet-tasting food are based in biological changes associated with pain. For instance, the experience of pain, including through cold pressor tasks such as that used in this investigation, is associated with increased cortisol, which could increase insulin and the desire to consume foods high in sugar (e.g., [41]).

These findings suggest that acute physical pain and social pain impact choices related to food consumption through different mechanisms. Social pain has been demonstrated to impact choice because it depletes regulatory resources (e.g., [19]). The present investigation reveals that this is not the case for physical pain. Of course, this was demonstrated with one manipulation that depletes regulatory resources and would need to be replicated with other manipulations, including those that reduce cognitive resources specifically. While this finding might at first appear inconsistent with models that physical and social pain share similar antecedents and consequences, most of those models have focused on chronic physical pain (e.g., [4]). Acute physical pain, the focus of the present investigation, is unlikely to trigger rumination, and thus to require cognitive resources, after its occurrence—once the pain ends, people generally stop thinking about it. In contrast, chronic physical pain is likely to continue to place a load on limited cognitive resources because it, by definition, does not end. Similarly, social pain, including acute social pain, often triggers rumination over the experience of rejection and therefore is likely to continue to load cognitive resources even after the end of the experience [19, 42]. Thus, the present findings also highlight an important distinction for models of the overlap between physical and social pain. Acute physical pain could have different consequences than acute social pain in any situation where the consequences are tied to the availability of regulatory resources.

That said, empirical evidence is steadily emerging that changed valuation of positive experiences can also affect consumption following acute social pain. Hayman, McIntyre, and Abbey [43] investigated the effects of social exclusion on emotional eating in African American women. Participants who were excluded based on race, compared to included participants, reported feeling more negative affect and consequently ate more potato chips. Similarly, Hales, Williams, and Eckhardt [44] tested whether alcohol intoxication dulls the pain and distress felt following acute social pain. The results indicated that alcohol intoxication reduced self-reported pain after exclusion. Furthermore, relying on stress-coping theory [45, 46], Gerrard and colleagues [47] demonstrated a relationship between social exclusion attributed to racial discrimination and increased willingness to use drugs or narcotic substances. Altogether these studies present a convincing pattern that links encounters of acute social pain to a desire to ingest experientially positive stimuli. Thus, in addition to reduced regulatory resources, social pain might impact consumption through changes in the hedonic valuation of positive experiences, as is the case with physical pain. Further work is needed to identify the mechanisms through which different types of pain influence choice.

The present investigation reveals that changes in the valuation of hedonically positive food can follow from painful experience, as reflected by increased consumption. People do attempt to eat the pain away, likely based on previous experiences with certain foods having an analgesic effect after painful experiences. Given the availability of sweet-tasting foods, this increased consumption after pain has potentially deleterious consequences for health, especially for those with chronic pain conditions.

## References

[1] World Health Organization (2013). Obesity and overweight. Retrieved from http://www.who.int/mediacentre/factsheets/fs311/en/

[2] Cawley J., & Meyerhoefer C. (2012). The medical care costs of obesity: An instrumental variables approach. Journal of Health Economics, 31, 219–230. 10.1016/j.jhealeco.2011.10.003 22094013

[3] Finkelstein E. A., DiBonaventuro M. D., Burgess S. M., & Hale B. C. (2010). The costs of obesity in the workplace. Journal of Occupational and Environmental Medicine, 52, 971–976. 10.1097/JOM.0b013e3181f274d2 20881629

[4] Riva P., Wesselmann E. D., Wirth J. H., Carter-Sowell A. R., & Williams K. D. (2014). When pain does not heal: The common antecedents and consequences of chronic social and physical pain. Basic and Applied Social Psychology, 36, 329–346.

[5] DeWall C. N., MacDonald G., Webster G. D., Masten C. L., Baumeister R. F., Powell C., Combs D., et al (2010). Acetaminophen reduces social pain: Behavioral and neural evidence. Psychological Science, 21, 931–937. 10.1177/0956797610374741 20548058

[6] Eisenberger N. I., Lieberman M. D., & Williams K. D. (2003). Does rejection hurt? An fMRI study of social exclusion. Science, 302, 290–292. 10.1126/science.1089134 14551436

[7] Miller R. R., & Grace R. C. (2003). Conditioning and learning. In Wiener I. B. (Ed. in Chief) and Healy A. F. & Proctor R. W. (Vol. Eds.), Handbook of psychology: Vol. 4. Experimental psychology (pp. 357–397). New York: Wiley.

[8] Baumeister R. F., DeWall C. N., Ciarocco N. J., & Twenge J. M. (2005). Social exclusion impairs self-regulation. Attitudes and Social Cognition, 88, 589–604. 10.1037/0022-3514.88.4.589 15796662

[9] MacDonald G., & Leary M. R. (2005). Why does social exclusion hurt? The relationship between social and physical pain. Psychological Bulletin, 131, 202–223. 10.1037/0033-2909.131.2.202 15740417

[10] Eisenberger N. I., & Lieberman M. D. (2004). Why rejection hurts: A common neural alarm system for physical and social pain. Trends in Cognitive Sciences, 8, 294–300. 10.1016/j.tics.2004.05.010 15242688

[11] Riva P., Wirth J. H., & Williams K. D. (2011). The consequences of pain: The social and physical pain overlap on psychological responses. European Journal of Social Psychology, 41, 681–687.

[12] Bickel W. K., Jarmolowicz D. P., Mueller E. T., Koffarnus M. N., & Gatchalian K. M. (2012). Pharmacology and Therapeutics, 134, 287–297. 10.1016/j.pharmthera.2012.02.004 22387232 PMC3329584

[13] Lench H. C. (2011). Personality and health outcomes: Making positive expectations a reality. Journal of Happiness Studies, 12, 493–507.

[14] Lench H. C., & Bench S. W. (2015). Strength of affective reaction as a signal to think carefully. Cognition and Emotion, 29, 220–235. 10.1080/02699931.2014.904223 24717008

[15] Greeno C. G., & Wing R. R. (1994). Stress-induced eating. Psychological Bulletin, 115, 444–464. 8016287 10.1037/0033-2909.115.3.444

[16] Tice D. M., Bratslavsky E., & Baumeister R. F. (2001). Emotional distress regulation takes precedence over impulse control: If you feel bad, do it! Journal of Personality and Social Psychology, 80, 53–67. 11195891

[17] Byrne K. A., Tibbett T. P., Laserna L. N., Carter-Sowell A. R., & Worthy D. A. (2015). Ostracism Reduces Reliance on Poor Advice from Others during Decision-Making. Journal of Behavioral Decision Making. 10.1002/bdm.1886 PMC5410892 28469290

[18] Baumeister R. F., Heatherton T. F., & Tice D. M. (1994). Losing Control: How and Why People Fail at Self-Regulation. San Diego, CA: Academic Press.

[19] Oaten M., Williams K. D., Jones A., & Zadro L. (2008). The effects of ostracism on self-regulation in the socially anxious. Journal of Social and Clinical Psychology, 27, 471–504.

[20] Apkarian A. V., Hashimi J. A., & Baliki M. N. (2011). Pain and the brain: Specificity and plasticity of the brain in clinical chronic pain. Pain, 152, 49–64. 10.1016/j.pain.2010.11.010 PMC3045648 21146929

[21] Flora S. R., Wilkerson L. R., & Flora D. B. (2003). Effects of cold pressor pain on human self-control for positive reinforcement. The Psychological Record, 53, 243–252.

[22] Rassu F., Furl B. & Meagher M. (2015). Impulsive decision making and experimental tonic pain. The Journal of Pain, 16, S49.

[23] Chen Z., Williams K. D., Fitness J., & Newton N. C. (2008). When hurt will not heal: Exploring the capacity to relive social and physical pain. Psychological Science, 19, 789–795. 10.1111/j.1467-9280.2008.02158.x 18816286

[24] Meyer M. L., Williams K. D., & Eisenberger N. I. (2015). Why social pain can live on: Different neural mechanisms are associated with reliving social and physical pain. PLOS One, 10(6), e0128294 10.1371/journal.pone.0128294 26061877 PMC4465485

[25] Hamilton N. A., Karoly P., & Kitzman H. (2004). Self-regulation and chronic pain: The role of emotion. Cognitive Therapy and Research, 28, 559–576.

[26] Drewnowski A., Krahn D. D, Demitrack M. A., Nairn K, & Gosnell B. (1995). Naloxone, an opiate blocker, reduces the consumption of sweet high-fat foods in obese and lean female binge eaters. American Journal of Clinical Nutrition, 61, 1206–1212. 7762518 10.1093/ajcn/61.6.1206

[27] Lewkowski M. D., Ditto B., Roussos M., & Young S. N. (2003). Pain, 106, 181–186. 14581126 10.1016/s0304-3959(03)00333-6

[28] Mercer M. E., & Holder M. D. (1997). Food cravings, endogenous opioid peptides, and food intake: A review. Appetite, 29, 325–352. 10.1006/appe.1997.0100 9468764

[29] Lewkowski M. D., Young S. N., Ghosh S., & Ditto B. (2008). Effects of opioid blockade on the modulation of pain and mood by sweet taste and blood pressure in young adults. Pain, 135, 75–81. 10.1016/j.pain.2007.05.003 17560720

[30] Gandhi W., Becker S. & Schweinhardt P. (2013). Pain increases drive to obtain reward, but does not affect associated hedonic responses: A behavioral study in healthy volunteers. European Journal of Pain, 17, 1093–1103. 10.1002/j.1532-2149.2012.00281.x 23349058

[31] Geha P., deAraujo B., Green B., & Small D. (2014). Decreased food pleasure and disrupted safety signals in chronic low back pain. Pain, 155, 712–722. 10.1016/j.pain.2013.12.027 24384160

[32] Kahneman D., & Tversky A. (1979). Prospect theory: An analysis of decision under risk. Econometrica, 47, 263–292.

[33] Walsh N. E., Schoenfeld L., Ramamurthy S., & Hoffman J. (1995). Normative model for cold pressor test. Annual Journal of Medical Rehabilitation, 68, 6–11. 10.1097/00002060-198902000-00003 2917058

[34] Wong D. L., & Baker C. M. (1988). Pain in children: Comparison of assessment scales. Pediatric Nursing, 14, 9–17. 3344163

[35] Muraven M., & Baumeister R. F. (2000). Self-regulation and depletion of limited resources: Does self-control resemble a muscle? Psychological Bulletin, 126, 247–259. 10748642 10.1037/0033-2909.126.2.247

[36] Tice D. M., Baumeister R. F., Shmueli D., & Muraven M. (2007). Restoring the self: Positive affect helps improve self-regulation following ego depletion. Journal of Experimental Social Psychology, 43, 379–384.

[37] Arnow B., Kenardy J., & Agras W. S. (1995). The Emotional Eating Scale: The development of a measure to assess coping with negative affect by eating. International Journal of Eating Disorders, 18, 79–90. 7670446 10.1002/1098-108x(199507)18:1<79::aid-eat2260180109>3.0.co;2-v

[38] Tangney J. P., Baumeister R. F., & Boone A. L. (2004). High self‐control predicts good adjustment, less pathology, better grades, and interpersonal success. Journal of Personality, 72, 271–324. 15016066 10.1111/j.0022-3506.2004.00263.x

[39] Gosling S. D., Rentfrow P. J., & Swann W. B. (2003). A very brief measure of the Big-Five personality domains. Journal of Research in Personality, 37, 504–528.

[40] American Academy of Pain Medicine (n. d.). AAPM Facts and Figures on Pain. Retrieved from http://www.painmed.org/patientcenter/facts_on_pain.aspx

[41] Adams T. C., & Epel E. S. (2007). Stress, eating and the reward system. Physiology and Behavior, 91, 449–458. 10.1016/j.physbeh.2007.04.011 17543357

[42] Wesselmann E. D., Ren D., Swim E., & Williams K. D. (2013). Rumination hinders recovery from ostracism. International Journal of Developmental Science, 7, 33–39.

[43] Hayman L. W., McIntyre R. B., & Abbey A. (2015). The bad taste of social ostracism: The effects of exclusion on the eating behaviors of African-American women. Psychology and Health, 30, 518–533. 10.1080/08870446.2014.983923 25403251

[44] Hales A. H., Williams K. D., & Eckhardt C. I. (2015). A participant walks into a bar…Subjective intoxication buffers ostracism’s negative effects. Social Psychology, 46, 157–166.

[45] Lazarus R. S., & Folkman S. (1984). Stress, Appraisal, and Coping. New York: Springer Publishing Company, Inc.

[46] Wills T. A. (1985). Stress, coping, and tobacco and alcohol use in early adolescence In Shiffman S & Wills T. A. (Eds.), Coping and Substance Abuse (pp. 67–94). Orlando, FL: Academic Press.

[47] Gerrard M., Stock M. L., Roberts M. E., Gibbons F. X, O’Hara R. E., Weng C. Y., et al (2012). Coping with racial discrimination: The role of substance use. Psychology of Addictive Behaviors, 26, 550–560. 10.1037/a0027711 22545585 PMC4079542

