# Correction: Do People Eat the Pain Away? The Effects of Acute Physical Pain on Subsequent Consumption of Sweet-Tasting Food

**DOI:** 10.1371/journal.pone.0173165

**Published:** 2017-02-24

**Authors:** Kathleen E. Darbor, Heather C. Lench, Adrienne R. Carter-Sowell

The following information is missing from the Funding section: The open access publishing fees for this article have been covered by the Texas A&M University Open Access to Knowledge Fund (OAKFund), supported by the University Libraries and the Office of the Vice President for Research.
